# DNA barcoding of native Caucasus herbal plants: potentials and limitations in complex groups and implications for phylogeographic patterns

**DOI:** 10.3897/BDJ.9.e61333

**Published:** 2021-01-27

**Authors:** Parvin Aghayeva, Salvatore Cozzolino, Donata Cafasso, Valida Ali-zade, Silvia Fineschi, Dilzara Aghayeva

**Affiliations:** 1 Institute of Botany, Azerbaijan National Academy of Sciences, Baku, Azerbaijan Institute of Botany, Azerbaijan National Academy of Sciences Baku Azerbaijan; 2 Department of Biology, University of Naples Federico II, Complesso Universitario di Monte S. Angelo, Napoli, Italy Department of Biology, University of Naples Federico II, Complesso Universitario di Monte S. Angelo Napoli Italy; 3 CNR - Istituto di Scienze del Patrimonio Culturale, Sesto Fiorentino, Italy CNR - Istituto di Scienze del Patrimonio Culturale Sesto Fiorentino Italy

**Keywords:** *accD*, asterids, Azerbaijan, barcoding identification, Caucasus, floristic surveys, *ITS*, native plants, orchids, *rpoB*

## Abstract

DNA barcoding has rapidly become a useful complementary tool in floristic investigations particularly for identifying specimens that lack diagnostic characters. Here, we assess the capability of three DNA barcode markers (chloroplast *rpoB*, *accD* and nuclear ITS) for correct species assignment in a floristic survey on the Caucasus. We focused on two herbal groups with potential for ornamental applications, namely orchids and asterids. On these two plant groups, we tested whether our selection of barcode markers allows identification of the “barcoding gap” in sequence identity and to distinguish between monophyletic species when employing distance-based methods. All markers successfully amplified most specimens, but we found that the rate of species-level resolution amongst selected markers largely varied in the two plant groups. Overall, for both lineages, plastid markers had a species-level assignment success rate lower than the nuclear ITS marker. The latter confirmed, in orchids, both the existence of a barcoding gap and that all accessions of the same species clustered together in monophyletic groups. Further, it also allowed the detection of a phylogeographic signal.The ITS marker resulted in its being the best performing barcode for asterids; however, none of the three tested markers showed high discriminatory ability. Even if ITS were revealed as the most promising plant barcode marker, we argue that the ability of this barcode for species assignment is strongly dependent on the evolutionary history of the investigated plant lineage.

## Introduction

DNA barcoding in botany has rapidly spread as a reliable tool for the accurate identification of plant species or genus (Hebert et al. 2004), as well as for determining the origin of plants and their derivatives (Galimberti et al. 2019, Saravanan et al. 2019). Several studies highlighted the potential ecological applications of DNA barcoding in biodiversity assessments of both existing and past communities (Valentini et al. 2009). It was largely utilised in studies on local floras and plant communities for identifying specimens that are hard to recognise by morphological characters or that lack diagnostic floral characters (such as rarely blooming species or species with a short blooming period and/or brief juvenile stages) including identification of cryptic species (Xu et al. 2018). DNA barcoding allows potentially higher levels of species discrimination, particularly at regional floristic level; in fact, a geographically-restricted context usually contains fewer closely-related species than a comprehensive taxonomic treatment (Kress et al. 2009). At a local scale, the approach is particularly reliable when combined with the development of localised barcoding libraries for determining the identity of unknown samples (Chase and Fay 2009, Kress et al. 2009). Accordingly, the capacity of DNA barcoding in resolving species in local floras has been tested in many plant groups, including species-rich tropical communities (Ebihara et al. 2010, Burgess et al. 2011, Costion et al. 2016). These studies have also demonstrated that combined chloroplast and nuclear markers provided additional discriminatory power and increased percentage of success in species-level assignment, compared to the more traditional two-locus (*rbc*L and *mat*K) barcode (Vijayan and Tsou 2010). Due to the high rate of nucleotide substitution, the relatively easy amplification and the large sequence data already available, the internal transcribed spacer (ITS) regions of the nuclear ribosomal cistron (18S-5.8S-26S) have been very successful at species-level discrimination across flowering plants (Li et al. 2011, Feng et al. 2016, Hosseinzadeh-Colagar et al. 2016a). Nuclear barcodes are particularly useful for cases of recent hybridisation or ongoing introgression, because they can recover different allelic variants from a sample (Chase and Fay 2009). Thus, nuclear markers have been usually combined with (haploid) plastid markers in most DNA barcoding studies (Hosseinzadeh-Colagar et al. 2016b, Castro et al. 2015). Indeed, the adaptation of a multi-locus barcoding system, with at least two markers, each representing a distinct DNA source as nuclear and organellar genome, could contribute to the gathering of independent evidence of the species attribution and accessions relationships from independent gene trees (Moore 1995, Hu et al. 2015). Finally, barcode markers may also eventually show consistent intra-specific variability (Hollingsworth et al. 2011). In that case and with a sampling representative of species distribution, haplotypic structure within a species can allow allocation of an individual to a geographic region and identify potential phylogeographic routes (Huemer and Hebert 2011).

The Caucasus represents one of the twenty-six biodiversity hotspot areas worldwide and has been the subject of botanical investigation since the beginning of the last century (Grossheim 1949, Karjagin 1928, Tutayuk et al. 1961). Recent research confirmed the Caucasus as part of the European flora (Bohn et al. 2007); indeed, many European plant lineages have close relatives in this region, including several domesticated plant species. The Caucasian flora represents a wonderful source of new food and medicinal plants and of new ornamentals with high adaptation potential in European gardens. Herbaceous monocots and dicots, particularly ornamental ones, are very numerous in the flora of the Caucasus and are characterised by low maintenance requirements. Indeed, these plants display high tolerance to environmental stresses as required for ornamental plants in the Mediterranean regions (Heywood 2003, Gray and Brady 2016).

Here, we employed DNA barcoding with the aim of investigating and quantifying plant diversity in the Quba and Qusar districts of Azerbaijan Caucasus. DNA-based methods are being increasingly used in floristic analyses, because they are not limited by taxonomic hindrances, such as: missing morphological features at any life stage (Wells and Stevens 2008, Ebihara et al. 2010); absence of distinctive identification characters in young or immature plants; and homoplasy of some characters (Vences et al. 2005). Nevertheless, the approach has some limitations when applied in the same critical groups as herbal species, particularly in the ability of species discrimination when closely-related species are examined (Chase and Fay 2009, Hubert and Hanner 2015). Here, we focused on two lineages, orchids and asterids, which are particularly interesting as they contain many ornamental species. We tested the potential of DNA barcodes for identifying unknown plant specimens and for identifying phylogenetic/phylogeographic relatedness with allied species and populations of other geographic origins. For this aim, we chose a combination of nuclear and plastid barcodes (ITS and chloroplast *rpoB*, *accD*), because DNA barcoding is particularly challenging when hybridisation might occur in conjunction with potential plastid capture or when lineage sorting has not yet been completed because of recent, rapid radiation (Fazekas et al. 2009, Chen et al. 2010), as expected in orchids and asterids. In particular, we tested whether the selection of barcode markers allows: i) the identification of the “barcoding gap” (Meyer and Paulay 2005) i.e. that the variation of the nucleotide sequences within species is lower than the differences amongst species and ii) the distinction between species, based on monophyletic clustering in distance-based neighbour-joining (NJ) trees (Hebert et al. 2004).

## Material and methods

**Study area.** Qusar and Quba districts are located between 500–4466 m above sea level in the in the south macro-slope of the Greater Caucasus and north-eastern part of Azerbaijan. These districts spread along various altitudinal zonations (foothills, low, middle and high mountain zones, subalpine, alpine habitats) and represent the richest floristic part of the country. The climate of the districts in summer is dry in the meadows and moderately hot in the foothills, whereas it is cold and very humid in the highlands and winter is usually cold. In the past couple of decades, increasing anthropogenic impacts, along with climate change, has contributed to the ecosystem degradation in these two districts.

**Sampling.** Approximately 500 ornamental herb specimens were collected during a floristic sampling campaign from 2012-2018 and were identified by means of morphological traits as belonging to 229 taxa, which are detailed as: 23 orders, 39 families and 129 genera. Morphologic identification was performed either by visual analysis or by using a dissection microscope, based on reliable diagnostic characters. Available checklists and recent literature on local floras (Karjagin 1928, Karjagin 1950, Aghayeva et al. 2018, Alizade et al. 2019, Tutayuk et al. 1961) were utilised as reference. The species status was further checked in the “World Flora Online” (http://www.worldfloraonline.org). Within this floristic survey, we selected altogether 54 accessions which were not clearly classified according to distinctive morphological characters. Thirty out of fifty-four accessions were roughly classified as Orchids and twenty-four accessions were roughly classified as Asterids. A small portion of leaf was preserved in silica gel and a barcode approach was performed as described below. All sampled specimens were collected in a herbarium within the Herbarium of the Institute of Botany, ANAS (BAK). We also had access to twenty herbarium vouchers of orchids and asterids, previously sampled from the same region and collected a small portion of dry specimens for performing the same barcode analysis.

**DNA isolation, amplification and sequencing**. Dried leaves from both field collection and herbarium samples were ground in a Tissue-lyser (Qiagen) and total DNA was extracted using GenElute™ Plant Genomic DNA Miniprep Kit (Sigma) following the manufacturer’s instructions. The nuclear ribosomal DNA (internal transcribed spacer regions ITS1 and ITS2) was amplified with primers described by Aceto et al. 1999). For plastid barcode analysis, the two coding regions *rpoB* (RNA polymerase subunit) and *accD* (acetyl-CoA carboxylase subunit) were amplified with specific primers (sequences available at http://www.kew.org; barcoding/protocol.htlm). All PCRs were performed in a final reaction volume of 25 µl using about 10 ng of template DNA, 200 mM of each dNTP, 10 pmol of each of the two primers, 1× *Taq* buffer (50 mM KCl, 10 mM Tris–HCl pH 9.0), 1.5 mM MgCl_2_ and 0.3 U of *Taq* polymerase (Sigma). Amplification of all barcodes was performed using the following protocol: initial denaturation at 94°C for 3 min, 35 cycles of denaturation at 94°C for 30 s, annealing at 53°C for 45 s and extension at 72°C for 1 min, followed by a final extension at 72°C for 7 min and final hold at 4°C. Amplification products were visualised on a 1.5% agarose gel and photographed after ethidium bromide staining. All successfully amplified DNA fragments were purified using the Clean Sweep PCR Purification Kit (Life Technology), following the manufacturer’s instructions and then sequenced in both directions using a modification of the Sanger dideoxy method as implemented in a double-stranded DNA cycle sequencing system with fluorescent dyes. Sequence reactions were then run on a 3130 Automated sequence system (Applied Biosystem).

Sequence editing and alignment were performed by using BioEdit v.7.2.0 (Hall 2018). The species discrimination ability of each barcode marker was evaluated using GenBank (http://www.ncbi.nlm.nih.gov), a public available nucleotide sequences database. For species assessment, the database was screened for the presence of each of the marker sequences at the species or genus level relative to our dataset, using BLAST (https://blast.ncbi.nlm.nih.gov/Blast.cgi). We considered as correct assignment when the query sequence has at least 99.5% of identical sites to the reference sequences in the database and when the top Bit-Score obtained in the GenBank matched the name of a single species. When the closest reference sequence scored lower than 99.5%, the result was considered as incomplete identification and imputable to the absence of the specific reference sequence in the database. Instead, when multiple reference sequences (i.e. from different species) shared the same top Bit-Score to the query sequence, the result was considered as incomplete identification due to insufficient discrimination power of the selected barcode.

Generated sequences and closest reference sequences (i.e. those identified by using BLAST and assigned to the same species) were aligned by using the MUSCLE programme in Mega X. For each barcode marker, a distance-based neighbour-joining (NJ) tree was then built with the Maximum Composite Likelihood model, uniform rates amongst sites and pairwise deletion in the gaps, for giving a graphic representation of the genetic distances within and amongst species.

## Data resources

Herbarium of the Institute of Botany, ANAS (BAK)

Dryad Data Repository - doi: 10.5061/dryad.2ngf1vhmw

## Results

In total, we examined 24 fresh samples and 14 herbarium vouchers for asterids and 30 fresh samples and six herbarium vouchers for orchids, respectively. We successfully amplified and sequenced all asterids, whereas two collected samples of the orchids dataset did not amplify with any marker and four other samples failed amplification across the three gene regions. Sequence recovery was slightly higher for plastid *rpoB* (88.8% samples) than for ITS (83.3% samples) markers (Tables 1, 2). All herbarium material from both plant lineages was successfully amplified and sequenced with selected barcode markers.

**Figure 1.**
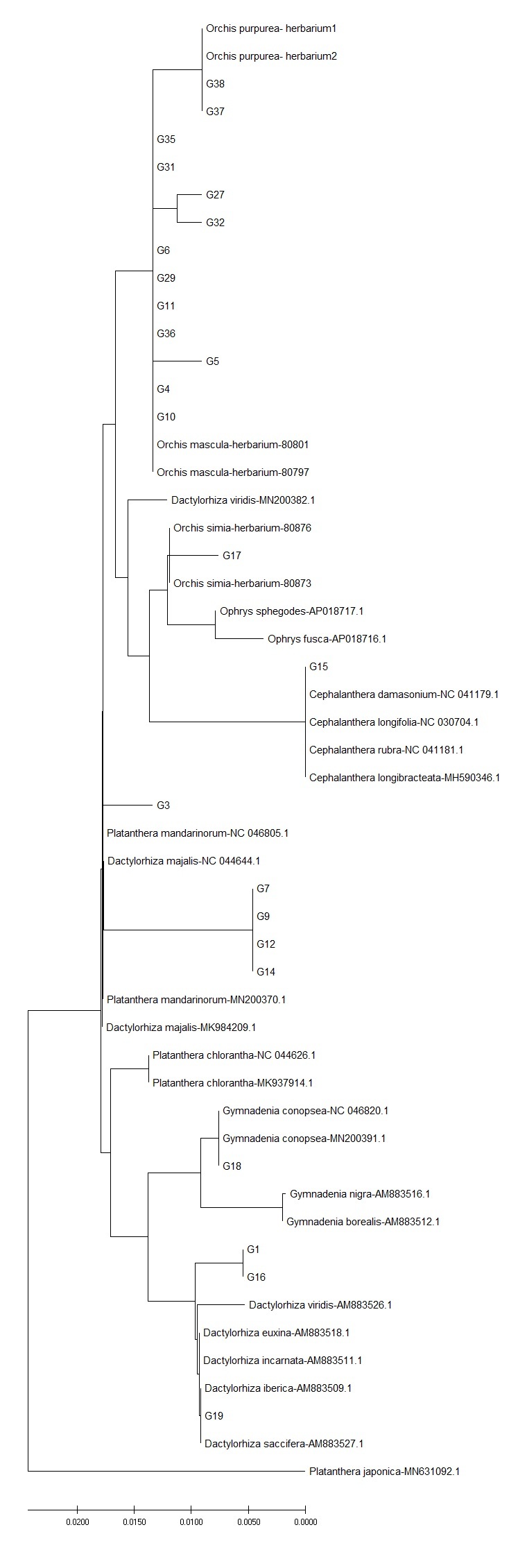
Neighbour-joining phylogenetic tree, based on *accD* sequences of selected orchids. All sequences have been deposited in the Dryad Data Repository - doi: 10.5061/dryad.2ngf1vhmw

**Figure 2.**
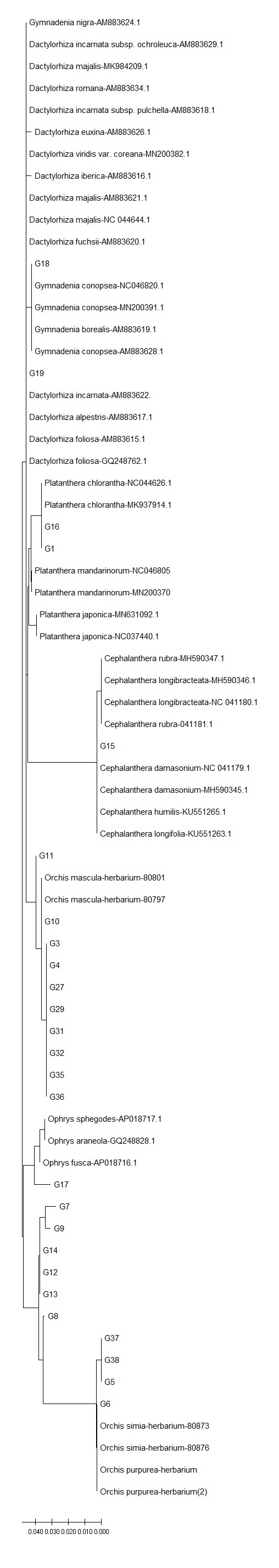
Neighbour-joining phylogenetic tree, based on *rpoB* sequences of selected orchids. All sequences have been deposited in the Dryad Data Repository - doi: 10.5061/dryad.2ngf1vhmw

**Figure 3.**
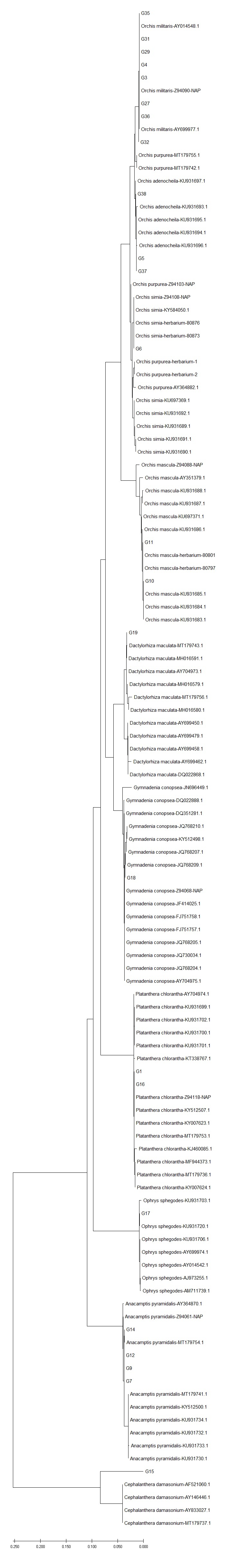
Neighbour-joining phylogenetic tree, based on ITS sequences of selected orchids. All sequences have been deposited in the Dryad Data Repository - doi: 10.5061/dryad.2ngf1vhmw

Local intraspecific variation for plastid barcodes was detected when multiple records were examined. In orchids, more than one haplotype for *accD* were detected in *O. purpurea* and *O. militaris* (Fig. 1) and different haplotypes for *rpoB* were detected in *O. mascula* and *A. pyramidalis* (Fig. 2). ITS base variation was detected in *O. mascula* and an ITS paralogy was detected in *O. purpurea* (Fig. 3).

**Figure 4.**
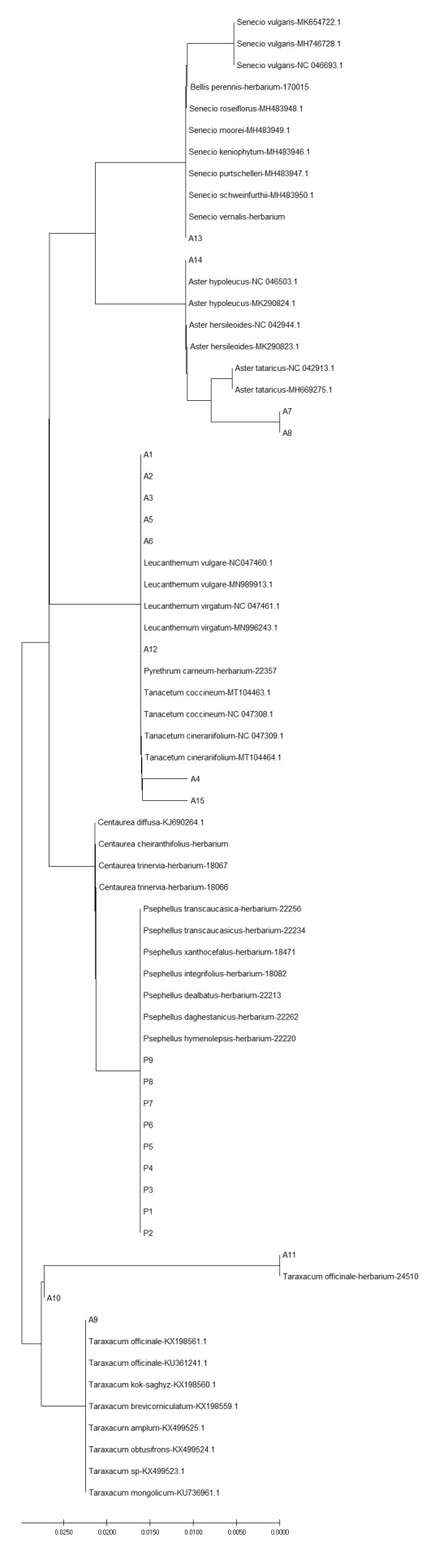
Neighbour-joining phylogenetic tree, based on *accD* sequences of selected asterids. All sequences have been deposited in the Dryad Data Repository - doi: 10.5061/dryad.2ngf1vhmw

**Figure 5.**
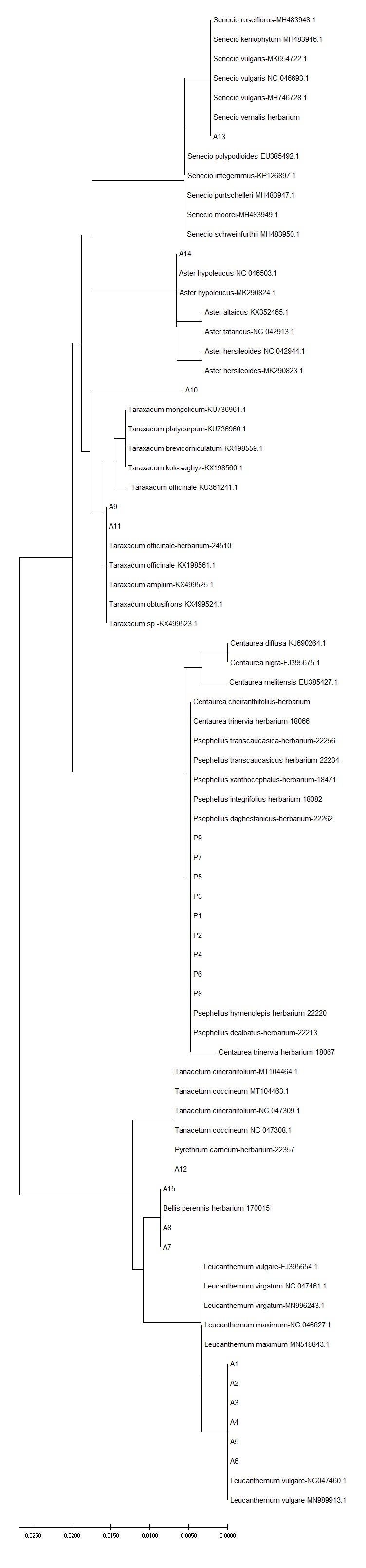
Neighbour-joining phylogenetic tree, based on *rpoB* sequences of selected asterids. All sequences have been deposited in the Dryad Data Repository - doi: 10.5061/dryad.2ngf1vhmw

**Figure 6.**
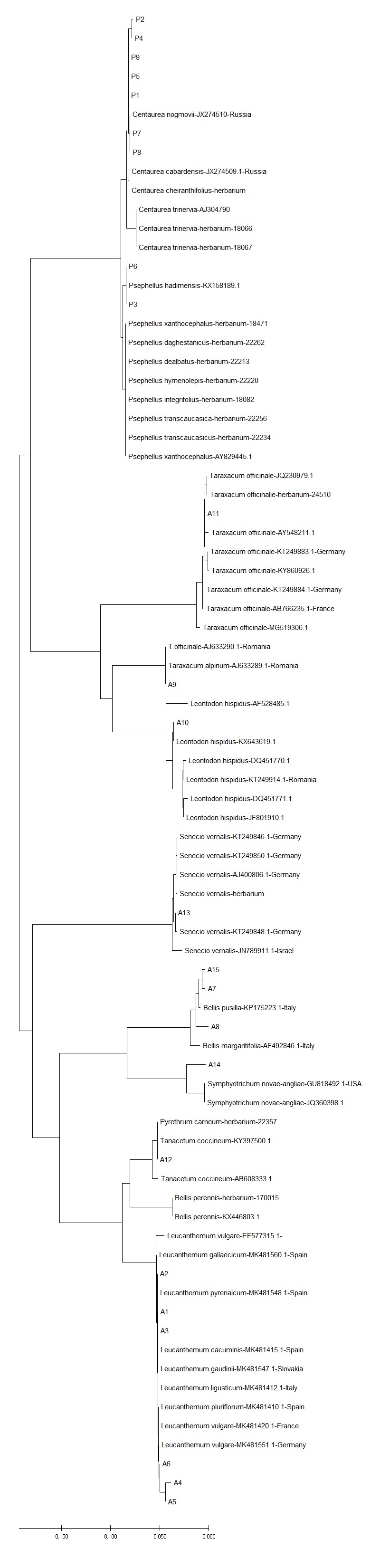
Neighbour-joining phylogenetic tree, based on ITS sequences of selected asterids. All sequences have been deposited in the Dryad Data Repository - doi: 10.5061/dryad.2ngf1vhmw

In asterids, variation for plastid *accD* was detected within genera (*Psephellus*, *Leucanthemum*), but not within species, with the notable exception of two haplotypes found in *Bellis perennis* (Fig. 4). No intraspecific and only very low interspecific variation (i.e. within genera) was detected for *rpoB* (Fig. 5). ITS variation within species was only detected between herbarium and wild-collected *Senecio vernalis* (Fig. 6).

Species discrimination ability using BLAST differs for each barcode marker and for the two plant groups. For orchids, ITS provided the highest species resolution (22 out of 26) (Table 3, Suppl. material 1), while both *accD* (1 out of 24) (Table 3, Suppl. material 2) and *rpoB* (3 out of 25) largely failed (Table 3, Suppl. material 3). For asterids, ITS (15 out of 24) (Table 4, Suppl. material 4) and *rpoB* (15 out of 24) (Table 4, Suppl. material 5) gave intermediate values for species resolution, while accD completely failed (Table 4, Suppl. material 6), as most species had identical sequences. Further, for asterids, there were several discrepancies on species assignment depending on the employed marker even when the top Bit-Score obtained in the GenBank matched a single species (in bold in Table 4).

**Figure 7.**
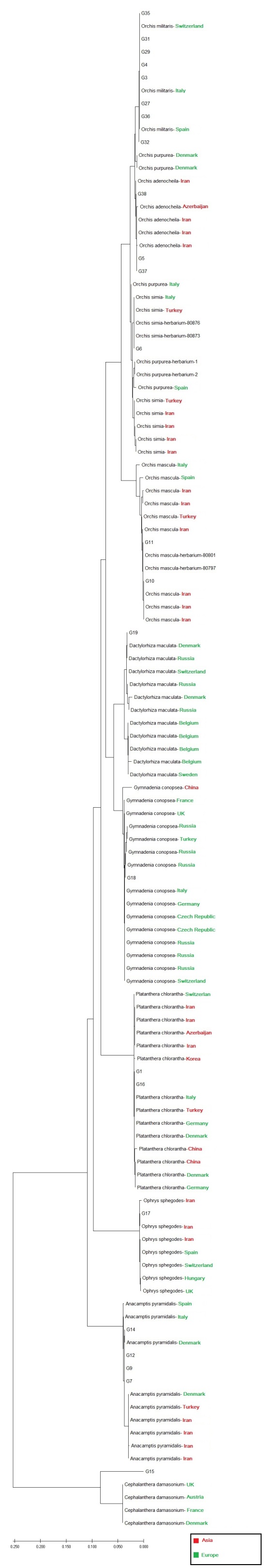
Neighbour-joining phylogenetic tree, based on *ITS* sequences of selected orchids with geographic origins (green: Europe; red: Asia) as inferred from Suppl. material 7.

**Figure 8.**
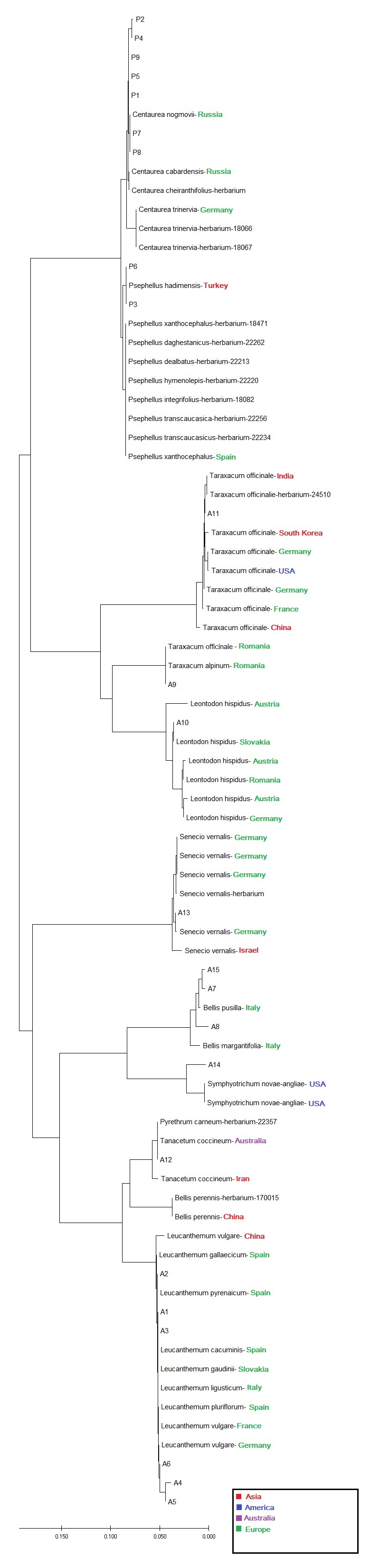
Neighbour-joining phylogenetic tree based, on *ITS* sequences of selected asterids with geographic origins (green: Europe; red: Asia) as inferred from Suppl. material 8.

ITS showed the highest discriminatory power also when evaluating genetic distances within and between species by NJ tree. This was evident in orchids: more than 90% of the sequences collected in this study had inter-specific diversity higher than intra-specific diversity, indicating that the ITS sequences had clear species boundaries and all accessions of the same species clustered in a monophyletic group (Table 5). Instead, in asterids, the discriminatory power of ITS marker was higher when discriminating amongst genera, but comparable with plastid markers when referring to species assignment (Table 6). When geographic origins of Genbank available sequences were plotted on the NJ tree, the ITS marker showed the phylogeographic signal for orchids (Fig. 7, Suppl. material 7), less for asterids (Fig. 8, Suppl. material 8). No phylogeographic variation was detected with plastid barcodes (data not shown).

## Discussion

We have tested the potential of barcode markers on a selection of herbal groups that are traditionally difficult to be morphologically identified since discriminant flower traits are not always available. Typically, a species discrimination is successful when the following conditions are met: i) all individual barcode sequences are not shared by any other species in the dataset; ii) genetic variation within species is lower than amongst species (i.e. the barcoding gap); iii) all individuals of a species cluster together in a monophyletic group when employing distance-based neighbour-joining (NJ) tree, at least at a local scale. Preliminary analyses of available information in public databases (GenBank) and literature data (Jin et al. 2014, Gao et al. 2010) confirmed the low level of species resolution when using traditional *rbcL* and *matK* barcodes in these two selected herbal groups. For this reason, we preferred testing complementary barcode markers, such as chloroplast *rpoB*, *accD* and nuclear ITS that are expected to have higher discriminatory power, particularly in annual/rapidly evolving herbaceous groups as the ones we were focused on (Chen et al. 2010, Gao et al. 2010). We chose these barcodes because of the sequence availability in public databases or, in the absence of available sequences, because of the level of interspecific variability detected with the same markers in related plant groups (Gigot et al. 2007, Dong et al. 2012).

We found that the selected barcodes successfully amplified and sequenced all asterids and almost all orchids (likely depending on the quality of dried samples, i.e. orchids have thicker leaves than asterids), but we found that the rate of species-level resolution largely varies amongst selected markers and plant groups. Overall, for both plant lineages, plastid markers had a species discrimination success rate lower than nuclear ITS, which allowed us, at least for orchids, to univocally discriminate most species. Sequence accessions of each species clustered together in monophyletic groups confirming the existence of a barcoding gap (Fig. 3). As already found in previous studies (Aceto et al. 1999, Cozzolino et al. 2001), variation found in the ITS region allows determination of genetic divergence amongst orchid species.

In orchids, ITS demonstrated a higher successful discrimination capability compared to both plastid markers, whereas in asterids, both ITS and *rpoB* had a comparable identification success (Table 4). *accD* completely fails in identifying asterids and most of orchids for both BLAST and the nearest genetic distance method. The lower identification success of plastid markers (particularly of *accD*) is largely due to its low discriminatory power (different species with identical sequences) or because of missing available reference sequences (Suppl. materials 2, 1, 3, 4, 5, 6, 7, 8).

In asterids, we also detected a discrepancy between species assignment with the query sequences (i.e. at least 99.5% of identical sites to reference sequences) for different barcodes. An example is given by the accession P6: ITS marker shared a top Bit-Score (100%) with the *Psephellus hadimensis* reference sequence, while the *rpoB* marker shared a top Bit-Score (100%) with the *Carthamus tinctorius* reference sequence (Table 4). Indeed, efficiency evaluation of correct assignment with DNA barcoding markers depends both on how informative are the generated sequences and how many sequences of representative groups are already available in public DNA databases. Ideally, the accuracy of specimen identification is highly dependent on representation of databases in which target species are represented by several individuals (Meyer and Paulay 2005) from different geographic origins. However, such databases are often not sufficiently complete and suited to exclude the risk of sequence matching due to missing data or of incorrect estimation of the barcoding gap (Meyer and Paulay 2005), as we found here, particularly for our plastid markers. In this perspective, the combined use of both unknown material and well-identified herbarium specimens, as implemented in our study, may partially fulfil such weaknesses (Kuzmina et al. 2017). Nevertheless, in case of discrepancy in the species assignment with different DNA barcoding markers, we preferred the assignment, based on those markers with larger bulk of reference sequences and/or that allow accessions to cluster monophyletically with distance-based approaches.

The discreteness of species boundaries, particularly in hybridising and/or fast-radiating lineages, may reduce the discriminatory power of barcode markers (Chase and Fay 2009, Kress et al. 2009). For this reason, the combined use of plastid and nuclear markers allows testing for hybridisation/reticulate evolution. In our case, we only detected a single case of ITS paralogy (in *O. purpurea*). Overall, we did not detect a discordantspecies relationship depending on the used barcode (nuclear or plastid) that could be a clear indication of hybridisation/reticulate evolution. This points to the low plastid marker resolution amongst closely-related species more likely due to their recent radiation (particularly in asterids). In that case, if barcode markers are evolving slowly, relative to the speciation rate, there may be insufficient nucleotide differences to distinguish recent species (Fazekas et al. 2009).

Barcode markers that univocally allow identifying species can also be used to reconstruct main phylogeographic patterns, if they contain enough intra-specific variability. In such cases, comparison of barcode sequences of plant specimens collected throughout their geographical ranges may provide sufficient informative data for allocating individuals to a well-defined geographic origin. Here, we also estimated whether nuclear and plastid markers were sufficiently variable to provide insights into the historical phylogeography and to detect the pattern of geographical distribution of infraspecific variation in Caucasian orchids and asterids. In our case, both plastid markers almost fail in identifying geographic origins of orchid and asterid accessions of different origins (identical barcode sequences) while ITS, at least for orchids, displayed enough infraspecific variation leading to different geographic rybotypes, potentially useful for tracking origins of plant materials.

Terrestrial orchids occurred both in the Caucasus and Europe. In particular, terrestrial Orchidinae probably originate from Irano-Turanian and Caucasus elements (the Irano-Turanian and Caucasus origin) and came into the Mediterranean basin during the Messinian age where their radiation gave rise to one of the richest systems of vicariant endemism between the two floristic regions. Some Mediterranean radiated lineages have then secondarily recolonised the Caucasian region (Batemann et al. 2003). Interestingly, for some orchid species (*Orchis mascula, Platanthera chloranta, Anacamptis pyramidalis*; Fig. 8), ITS sequences clearly display such geographic variation (from west to east and vice versa), while, for other species, almost no sequence variation occurs across all ranges (*Orchis militaris)*. In the former case, we suggest that this intraspecific variation represents the signature of ancient phylogeographic routes, whereas in the latter, with no intraspecific variation, we suspect recent post glacial phylogeographic migration erased the ancient phylogeographic signal.

## Conclusions

We found, for both lineages, plastid markers had a species-level assignment success rate lower that nuclear ITS marker. Several processes, such as recent speciation events with incomplete lineage sorting and retention of ancestral sequences, may cause a partial failure of DNA barcodes to track species events. Indeed, the ITS sequence was successful in orchids, but not in many asterids. We argue that, at least between the two herbal groups, the diversification time marked the difference in barcode efficiency as the absence of a barcoding gap amongst closely-related, recently-diverged species is quite common. While orchids represent an old evolutionary lineage, with some groups radiating in the Mediterranean and secondarily migrating to the Caucasus (Batemann et al. 2003), diversification of asterid lineages is more recent and had its centre in the Caucasus and surrounding west Asia (Barres et al. 2013). In contrast to orchids, many closely-related asterids species occur within a geographically-restricted context, which makes difficult their discrimination, particularly with plastid barcodes. Overall, our study suggests that the ITS sequence can be potentially utilised as universal plant barcodes in herbal groups; at the same time, it highlights that ITS sequence efficiency as barcode marker and its discriminatory power are strongly dependent on the evolutionary history of the examined plant group.

## Supplementary Material

7C37DF02-4122-5C0D-BAD7-A2B209DFD7D5 10.3897/BDJ.9.e61333.suppl1 Supplementary material 1 Results of BLAST species identification test for orchid ITS Data type Top Bit-score Brief description BLAST identification for orchid File: oo_447834.docx https://binary.pensoft.net/file/447834 Parvin Aghayeva, Salvatore Cozzolino, Donata Cafasso, Valida Ali-zade, Silvia Fineschi, Dilzara Aghayeva

D3018B5F-291A-5881-B057-D1DC734A7D2A 10.3897/BDJ.9.e61333.suppl2 Supplementary material 2 Results of BLAST species identification test for orchid *accD* Data type Top Bit-score Brief description BLAST identification for orchid File: oo_447827.docx https://binary.pensoft.net/file/447827 Parvin Aghayeva, Salvatore Cozzolino, Donata Cafasso, Valida Ali-zade, Silvia Fineschi, Dilzara Aghayeva

FC657A1B-E9A2-5D56-B960-4609268C61C8 10.3897/BDJ.9.e61333.suppl3 Supplementary material 3 Results of BLAST species identification test for orchid *rpoB* Data type Top Bit-score Brief description BLAST identification for orchid File: oo_447828.docx https://binary.pensoft.net/file/447828 Parvin Aghayeva, Salvatore Cozzolino, Donata Cafasso, Valida Ali-zade, Silvia Fineschi, Dilzara Aghayeva

307E06C6-F30D-5337-B167-C9D334B95D24 10.3897/BDJ.9.e61333.suppl4 Supplementary material 4 Results of BLAST species identification test for asterid ITS Data type Top Bit-score Brief description BLAST identification for asterid File: oo_447830.docx https://binary.pensoft.net/file/447830 Parvin Aghayeva, Salvatore Cozzolino, Donata Cafasso, Valida Ali-zade, Silvia Fineschi, Dilzara Aghayeva

BDBD31D0-50DD-5064-8292-C9DBB5CD5141 10.3897/BDJ.9.e61333.suppl5 Supplementary material 5 Results of BLAST species identification test for asterid *rpoB* Data type Top Bit-score Brief description BLAST identification for asterid File: oo_447832.docx https://binary.pensoft.net/file/447832 Parvin Aghayeva, Salvatore Cozzolino, Donata Cafasso, Valida Ali-zade, Silvia Fineschi, Dilzara Aghayeva

4D110467-A84A-56EC-998F-628EFABC59C4 10.3897/BDJ.9.e61333.suppl6 Supplementary material 6 Results of BLAST species identification test for asterid *accD* Data type Top Bit-score Brief description BLAST identification for asterid File: oo_452656.docx https://binary.pensoft.net/file/452656 Parvin Aghayeva, Salvatore Cozzolino, Donata Cafasso, Valida Ali-zade, Silvia Fineschi, Dilzara Aghayeva

8143F9FB-DF24-5C88-8FAD-3E11AA1CF35D 10.3897/BDJ.9.e61333.suppl7 Supplementary material 7 Highest BLAST match of ITS sequences for the samples of Orchidaceae examined in this study Data type BLAST match Brief description BLAST match of ITS sequences for Orchidaceae File: oo_447845.doc https://binary.pensoft.net/file/447845 Parvin Aghayeva, Salvatore Cozzolino, Donata Cafasso, Valida Ali-zade, Silvia Fineschi, Dilzara Aghayeva

D44D2CC4-0321-5DC6-9E7B-120AAF0FE34F 10.3897/BDJ.9.e61333.suppl8 Supplementary material 8 Highest BLAST match of ITS sequences for the samples of Asteraceae examined in this study Data type BLAST match Brief description BLAST match of ITS of Asteraceae File: oo_447846.doc https://binary.pensoft.net/file/447846 Parvin Aghayeva, Salvatore Cozzolino, Donata Cafasso, Valida Ali-zade, Silvia Fineschi, Dilzara Aghayeva

## Figures and Tables

**Table 1.** Sequence recovery for the three selected barcode regions from unknown (G1-G38) and Herbarium orchid samples.

Sample	*ITS*		*rpoB*		*accD*	
	amplification	sequencing	amplification	sequencing	amplification	sequencing
G1	x	x	x	x	x	x
G3	x	x	x	x	x	x
G4	x	x	x	x	x	x
G5	x	x	x	x	x	x
G6	x	x	x	x	x	x
G7	x	x	x	x	x	x
G8	NO	NO	x	x	x	NO
G9	x	x	x	x	x	x
G10	x	x	x	x	x	x
G11	x	x	x	x	x	x
G12	x	x	x	x	x	x
G13	NO	NO	x	x	x	NO
G14	x	x	x	x	x	x
G15	x	x	x	x	x	x
G16	x	x	x	x	x	x
G17	x	x	x	x	x	x
G18	x	x	x	x	x	x
G19	x	x	x	x	x	x
G27	x	x	x	x	x	x
G28	NO	NO	NO	NO	NO	NO
G29	x	x	x	x	x	x
G30	NO	NO	NO	NO	NO	NO
G31	x	x	x	x	x	x
G32	x	x	x	x	x	x
G33	NO	NO	x	NO	x	NO
G34	NO	NO	x	NO	x	NO
G35	x	x	x	x	x	x
G36	x	x	x	x	x	x
G37	x	x	x	x	x	x
G38	x	x	x	x	x	x
*Orchis purpurea* Herbarium 1	x	x	x	x	x	x
*Orchis purpurea* Herbarium 2	x	x	x	x	x	x
*Orchis simia* Herbarium 80873	x	x	x	x	x	x
*Orchis simia* Herbarium 80876	x	x	x	x	x	x
*Orchis mascula* Herbarium 80801	x	x	x	x	x	x
*Orchis mascula* Herbarium 80797	x	x	x	x	x	x
	30/36 83.3%		32/36 88.8%		30/36 83.3%	

**Table 2.** Sequence recovery for the three selected barcode regions from unknown (P1-A15) and Herbarium asterid samples.

Sample	*ITS*		*rpoB*		*accD*	
	amplification	sequencing	amplification	sequencing	amplification	sequencing
P1	x	x	x	x	x	x
P2	x	x	x	x	x	x
P3	x	x	x	x	x	x
P4	x	x	x	x	x	x
P5	x	x	x	x	x	x
P6	x	x	x	x	x	x
P7	x	x	x	x	x	x
P8	x	x	x	x	x	x
P9	x	x	x	x	x	x
A1	x	x	x	x	x	x
A2	x	x	x	x	x	x
A3	x	x	x	x	x	x
A4	x	x	x	x	x	x
A5	x	x	x	x	x	x
A6	x	x	x	x	x	x
A7	x	x	x	x	x	x
A8	x	x	x	x	x	x
A9	x	x	x	x	x	x
A10	x	x	x	x	x	x
A11	x	x	x	x	x	x
A12	x	x	x	x	x	x
A13	x	x	x	x	x	x
A14	x	x	x	x	x	x
A15	x	x	x	x	x	x
*Centaurea trinervia* Herbarium 18066	x	x	x	x	x	x
*Centaurea trinervia* Herbarium 18067	x	x	x	x	x	x
*Psephellus hymenolepis* Herbarium 22220	x	x	x	x	x	x
*Psephellus daghestanicus* Herbarium 22262	x	x	x	x	x	x
*Psephellus dealbatus* Herbarium 22213	x	x	x	x	x	x
*Psephellus intergrifolius* Herbarium 18082	x	x	x	x	x	x
*Psephellus xantocephalus* Herbarium 18471	x	x	x	x	x	x
*Psephellus transcaucasicus* Herbarium 22234	x	x	x	x	x	x
*Psephellus transcaucasicus* Herbarium 22256	x	x	x	x	x	x
*Pyrethrum carneum* Herbarium 22357	x	x	x	x	x	x
*Taraxacum officinale* Herbarium 24510	x	x	x	x	x	x
*Senecio vernalis* Herbarium	x	x	x	x	x	x
*Bellis perennis* Herbarium 170015	x	x	x	x	x	x
*Centaurea cheiranthifolius* Herbarium	x	x	x	x	x	x
	38/38 100%		38/38 100%		38/38 100%	

**Table 3.** Orchid species resolution for each barcode region, based on an all-to-all Blast analysis. NO^[1]^: more than one reference sequence at top Bit-Score (at least 99.5%) NO^[2]^: all reference sequences at top Bit-score lower than 99.5%

Sample	*ITS*	*accD*	*rpoB*
G1	NO^[1]^	NO^[1]^	(*Platanthera chlorantha*)
G3	(*Orchis militaris*)	NO^[1]^	NO ^[2]^
G4	(*Orchis militaris*)	NO^[1]^	NO ^[2]^
G5	(*Orchis adenocheila*)	NO^[2]^	NO ^[2]^
G6	(*Orchis simia*)	NO^[1]^	NO ^[2]^
G7	*(Anacamptis pyramidalis*)	NO^[2]^	NO ^[2]^
G8			NO ^[2]^
G9	(*Anacamptis pyramidalis*)	NO^[2]^	NO^[2]^
G10	(*Orchis mascula*)	NO ^[1]^	NO^[2]^
G11	(*Orchis mascula*)	NO ^[1]^	NO^[2]^
G12	(*Anacamptis pyramidalis*)	NO^[2]^	NO^[2]^
G13			NO ^[2]^
G14	(*Anacamptis pyramidalis*)	NO^[2]^	NO^[2]^
G15	NO^[1]^	NO^[1]^	NO^[1]^
G16	NO^[1]^	NO^[1]^	(*Platanthera chlorantha*)
G17	(*Ophrys sphegodes*)	NO^[2]^	NO^[2]^
G18	(*Gymnadenia conopsea*)	NO^[1]^	(*Gymnadenia conopsea*)
G19	NO^[1]^	(*Dactylorhiza saccifera*)	NO^[1]^
G27	(*Orchis militaris*)	NO^[2]^	NO^[2]^
G29	(*Orchis militaris*)	NO^[1]^	NO ^[2]^
G31	(*Orchis militaris*)	NO^[2]^	NO^[2]^
G32	(*Orchis militaris*)	NO^[2]^	NO^[2]^
G35	(*Orchis militaris*)	NO^[2]^	NO^[2]^
G36	(*Orchis militaris*)	NO^[1]^	NO ^[2]^
G37	(*Orchis adenocheila*)	NO^[2]^	NO^[2]^
G38	(*Orchis adenocheila*)	NO^[2]^	NO^[2]^

**Table 4.** Asterid species resolution for each barcode region, based on an all-to-all Blast analysis. NO^[1]^: more than one reference sequence at top Bit-Score (at least 99.5%) NO^[2]^: all reference sequences at top Bit-score lower than 99.5%

Sample	*ITS*	*accD*	*rpoB*
**P1**	**(*Centaurea nogmovii*)**	NO ^[1]^	**(*Carthamus tinctorius*)**
**P2**	NO ^[2]^	NO ^[1]^	(*Carthamus tinctorius*)
**P3**	**(*Psephellus hadimensis*)**	NO ^[1]^	**(*Carthamus tinctorius*)**
**P4**	**(*Centaurea nogmovii*)**	NO ^[1]^	**(*Carthamus tinctorius*)**
**P5**	**(*Centaurea nogmovii*)**	NO ^[1]^	**(*Carthamus tinctorius*)**
**P6**	**(*Psephellus hadimensis*)**	NO ^[1]^	**(*Carthamus tinctorius*)**
**P7**	**(*Centaurea nogmovii*)**	NO ^[1]^	**(*Carthamus tinctorius*)**
**P8**	**(*Centaurea nogmovii*)**	NO ^[1]^	**(*Carthamus tinctorius*)**
**P9**	**(*Centaurea nogmovii*)**	NO ^[1]^	**(*Carthamus tinctorius*)**
**A1**	NO^[1]^	NO ^[1]^	(*Leucanthemum vulgare*)
**A2**	NO ^[2]^	NO ^[1]^	(*Leucanthemum vulgare*)
**A3**	NO ^[1]^	NO ^[1]^	(*Leucanthemum vulgare*)
**A4**	NO^[2]^	NO ^[1]^	(*Leucanthemum vulgare*)
**A5**	NO^[1]^	NO ^[1]^	(*Leucanthemum vulgare*)
**A6**	NO ^[2]^	NO ^[1]^	(*Leucanthemum vulgare*)
**A7**	(*Bellis pusilla*)	NO ^[2]^	NO^[1]^
**A8**	(*Bellis pusilla*)	NO ^[2]^	NO^[1]^
**A9**	(*Hypochaeris radicata*)	NO^[1]^	NO^[1]^
**A10**	(*Leontodon hispidus*)	NO^[1]^	NO^[1]^
**A11**	NO ^[1]^	NO ^[2]^	NO^[1]^
**A12**	(*Tanacetum coccineum*)	NO^[1]^	NO^[1]^
**A13**	(*Senecio vernalis*)	NO^[1]^	NO^[1]^
**A14**	(*Symphyotrichum novae-angliae*)	NO^[1]^	NO^[1]^
**A15**	NO^[2]^	NO^[1]^	NO^[1]^

**Table 5.** Orchid species resolution for each barcode region, based on the NJ tree (i.e. monophyletic species)

Sample	*ITS*	*accD*	*rpoB*
**G1**	(*Platanthera chlorantha*)	NO	(*Platanthera chlorantha*)
**G3**	(*Orchis militaris*)	NO	NO
**G4**	(*Orchis militaris*)	NO	NO
**G5**	(*Orchis adenocheila*)	NO	NO
**G6**	(*Orchis simia*)	NO	NO
**G7**	(*Anacamptis pyramidalis*)	NO	NO
**G9**	(*Anacamptis pyramidalis*)	NO	NO
**G10**	(*Orchis mascula*)	NO	(*Orchis mascula*)
**G11**	(*Orchis mascula*)	NO	NO
**G12**	(*Anacamptis pyramidalis*)	NO	NO
**G14**	(*Anacamptis pyramidalis*)	NO	NO
**G15**	NO (*Cephalanthera* sp.)	NO (*Cephalanthera* sp.)	NO (*Cephalanthera* sp.)
**G16**	(*Platanthera chlorantha*)	NO	(*Platanthera chlorantha)*
**G17**	(*Ophrys sphegodes*)	(*Orchis simia*)	NO (*Ophrys* sp.)
**G18**	(*Gymnadenia conopsea*)	(*Gymnadenia conopsea*)	(*Gymnadenia conopsea*)
**G19**	(*Dactylorhiza maculata*)	NO (*Dactylorhiza* sp.)	NO (*Dactylorhiza* sp.)
**G27**	*Orchis militaris*	NO	NO
**G29**	(*Orchis militaris*)	NO	NO
**G31**	(*Orchis militaris*)	NO	NO
**G32**	(*Orchis militaris*)	NO	NO
**G35**	(*Orchis militaris*)	NO	NO
**G36**	(*Orchis militaris*)	NO	NO
**G37**	(*Orchis adenocheila*)	(*Orchis purpurea*)	NO
**G38**	(*Orchis adenocheila*)	(*Orchis purpurea*)	NO
**G13**			NO
**G8**			NO

**Table 6.** Asterid species resolution for each barcode region, based on the NJ tree (i.e. monophyletic species).

Sample	*ITS*	*accD*	*rpoB*
**P1**	NO (*Centaurea* sp.)	NO (*Psephellus* sp.)	NO (*Psephellus* sp.)
**P2**	NO	NO (*Psephellus* sp.)	NO (*Psephellus* sp.)
**P3**	(*Psephellus hadimensis*)	NO (*Psephellus* sp.)	NO (*Psephellus* sp.)
**P4**	NO (*Centaurea* sp.)	NO (*Psephellus* sp.)	NO (*Psephellus* sp.)
**P5**	NO (*Centaurea* sp.)	NO (*Psephellus* sp.)	NO (*Psephellus* sp.)
**P6**	(*Psephellus hadimensis*)	NO (*Psephellus* sp.)	NO (*Psephellus* sp.)
**P7**	(*Centaurea nogmovii*)	NO (*Psephellus* sp.)	NO (*Psephellus* sp.)
**P8**	(*Centaurea nogmovii*)	NO (*Psephellus* sp.)	NO (*Psephellus* sp.)
**P9**	NO (*Centaurea* sp.)	NO (*Psephellus* sp.)	NO (*Psephellus* sp.)
**A1**	NO (*Leucanthemum* sp.)	NO (*Leucanthemum* sp.)	(*Leucanthemum vulgare*)
**A2**	NO (*Leucanthemum* sp.)	NO (*Leucanthemum* sp.)	(*Leucanthemum vulgare*)
**A3**	NO (*Leucanthemum* sp.)	NO (*Leucanthemum* sp.)	(*Leucanthemum vulgare*)
**A4**	NO	NO	(*Leucanthemum vulgare*)
**A5**	NO	NO (*Leucanthemum* sp.)	(*Leucanthemum vulgare*)
**A6**	NO (*Leucanthemum* sp.)	NO (*Leucanthemum* sp.)	(*Leucanthemum vulgare*)
**A7**	(*Bellis pusilla*)	NO	(*Bellis perennis*)
**A8**	NO (*Bellis* sp.)	NO	(*Bellis perennis*)
**A9**	NO (*Taraxacum* sp.)	NO (*Taraxacum* sp.)	NO (*Taraxacum* sp.)
**A10**	(*Leontodon hispidus)*	NO	NO
**A11**	(*Taraxacum officinale)*	*(Taraxacum Officinale*)	NO (*Taraxacum* sp.)
**A12**	(*Tanacetum coccineum*)	NO	NO
**A13**	(*Senecio vernalis*)	NO (*Senecio* sp.)	NO (*Senecio* sp.)
**A14**	NO	NO (*Aster* sp.)	(*Aster hypoleucus*)
**A15**	NO	NO	(*Bellis perennis*)
