# Supplementary material for: DNA barcoding of native Caucasus herbal plants: potentials and limitations in complex groups and implications for phylogeographic patterns
Source: Biodivers Data J. 2021 Jan 27;9:e61333. doi: 10.3897/BDJ.9.e61333 (PMC7858560; doi:10.3897/BDJ.9.e61333)
Supplement: Supplementary material 1 — Results of BLAST species identification test for orchid ITS [file bdj-09-e61333-s001.docx]

SuppTab1: Results of BLAST species identification test for orchid ITS

| Sample | Top Bit-score |
| --- | --- |
| ***G1*** | *Platanthera chlorantha* 100%  *Platanthera bifolia* 100% **NO^[1]^** |
| ***G3*** | *Orchis militaris* 100%  *Orchis purpurea* 97.97% **YES** |
| ***G4*** | *Orchis militaris* 100%  *Orchis purpurea* 97.97% **YES** |
| ***G5*** | *Orchis adenocheila* 99.84%  *Orchis purpurea* 99.06% **YES** |
| ***G6*** | *Orchis simia* 100%  *Orchis purpurea* 99.22% **YES** |
| ***G7*** | *Anacamptis pyramidalis* 100%  *Anacamptis coriophora* 92.25% **YES** |
| ***G9*** | *Anacamptis pyramidalis* 99.52%  *Anacamptis coriophora* 92.25% **YES** |
| ***G10*** | *Orchis mascula* 100%  *Orchis provincialis* 97.20% **YES** |
| ***G11*** | *Orchis mascula* 99.84%  *Orchis provincialis* 97.36% **YES** |
| ***G12*** | *Anacamptis pyramidalis* 99.52%  *Anacamptis coriophora* 92.25% **YES** |
| ***G14*** | *Anacamptis pyramidalis* 99.52%  *Anacamptis coriophora* 92.25% **YES** |
| ***G15*** | *Cephalanthera kotschyana* 100%  *Cephalanthera damasonium* 100%  *Cephalanthera longifolia* 97.88% **NO^[1]^** |
| ***G16*** | *Platanthera chlorantha* 100%  *Platanthera bifolia* 100% **NO^[1]^** |
| ***G17*** | *Ophrys sphegodes* 100%  *Ophrys cretica* 99.84% **YES** |
| ***G18*** | *Gymnadenia conopsea* 99.84%  *Gymnadenia odoratissima* 99.53% **YES** |
| ***G19*** | *Dactylorhiza maculata* 99.53%  *Dactylorhiza fuchsii* 99.53% **NO^[1]^** |
| ***G27*** | *Orchis militaris* 100%  *Orchis purpurea* 97.97% **YES** |
| ***G29*** | *Orchis militaris* 100%  *Orchis purpurea* 97.97% **YES** |
| ***G31*** | *Orchis militaris* 100%  *Orchis purpurea* 97.97% **YES** |
| ***G32*** | *Orchis militaris* 99.84%  *Orchis purpurea* 97.97% **YES** |
| ***G35*** | *Orchis militaris* 100%  *Orchis purpurea* 97.97% **YES** |
| ***G36*** | *Orchis militaris* 100%  *Orchis purpurea* 97.97% **YES** |
| ***G37*** | *Orchis adenocheila* 99.84%  *Orchis purpurea* 99.06% **YES** |
| ***G38*** | *Orchis adenocheila* 99.53%  *Orchis purpurea* 98.75% **YES** |

NO^[1]^: more than one reference sequence at top Bit-Score (at least 99.5 %)

NO^[2]^: all reference sequences at top Bit-score lower than 99.5%
