# Supplementary material for: DNA barcoding of native Caucasus herbal plants: potentials and limitations in complex groups and implications for phylogeographic patterns
Source: Biodivers Data J. 2021 Jan 27;9:e61333. doi: 10.3897/BDJ.9.e61333 (PMC7858560; doi:10.3897/BDJ.9.e61333)
Supplement: Supplementary material 2 — Results of BLAST species identification test for orchid accD [file bdj-09-e61333-s002.docx]

SuppTab2: Results of BLAST species identification test for orchid *accD*

| Sample | Top Bit-score |
| --- | --- |
| **G1** | *Dactylorhiza euxina* 99.57%  *Dactylorhiza incarnata* 99.57%  **NO^[1]^** |
| **G3** | *Dactylorhiza majalis* 99.57%  *Platanthera mandarinorum* 99.57%  **NO^[1]^** |
| **G4** | *Dactylorhiza majalis* 99.57%  *Platanthera mandarinorum* 99.57%  **NO^[1]^** |
| **G5** | *Dactylorhiza majalis* 99.14%  *Platanthera mandarinorum* 99.14%  **NO^[2]^** |
| **G6** | *Dactylorhiza majalis* 99.57%  *Platanthera mandarinorum* 99.57%  **NO^[1]^** |
| **G7** | *Dactylorhiza majalis* 98.71%  *Platanthera mandarinorum* 98.71%  **NO^[2]^** |
| **G9** | *Dactylorhiza majalis* 98.71%  *Platanthera mandarinorum* 98.71%  **NO^[2]^** |
| **G10** | *Platanthera mandarinorum* 99.57%  *Dactylorhiza majalis* 99.57%  **NO^[1]^** |
| **G11** | *Platanthera mandarinorum* 99.57%  *Dactylorhiza majalis* 99.57%  **NO^[1]^** |
| **G12** | *Dactylorhiza majalis* 98.71%  *Platanthera mandarinorum* 98.71%  **NO^[2]^** |
| **G14** | *Dactylorhiza majalis* 98.71%  *Platanthera mandarinorum* 98.71%  **NO^[2]^** |
| **G15** | *Epipactis thunbergii* 100%  *Cephalanthera rubra* 100%  **NO^[1]^** |
| **G16** | *Dactylorhiza euxina* 99.57%  *Dactylorhiza incarnata* 99.57%  **NO^[1]^** |
| **G17** | *Dactylorhiza majalis* 99.14%  *Ponerorchis gracilis* 99.14%  **NO^[2]^** |
| **G18** | *Gymnadenia conopsea* 100%  *Galearis cyclochila* 100%  **NO^[1]^** |
| **G19** | *Dactylothiza saccifera* 99.57%  *Dactylorhiza euxina* 97.85% **YES** |
| **G27** | *Dactylorhiza majalis* 99.14%  *Platanthera mandarinorum* 99.14%  **NO^[2]^** |
| **G29** | *Dactylorhiza majalis* 99.57%  *Platanthera mandarinorum* 99.57%  **NO^[1]^** |
| **G31** | *Dactylorhiza majalis* 99.15%  *Platanthera mandarinorum* 99.15%  **NO^[2]^** |
| **G32** | *Dactylorhiza majalis* 98.72%  *Platanthera mandarinorum* 98.72%  **NO^[2]^** |
| **G35** | *Dactylorhiza majalis* 99.15%  *Platanthera mandarinorum* 99.15%  **NO^[2]^** |
| **G36** | *Dactylorhiza majalis* 99.57%  *Platanthera mandarinorum* 99.57%  **NO^[1]^** |
| **G37** | *Dactylorhiza majalis* 98.71%  *Platanthera mandarinorum* 98.71%  **NO^[2]^** |
| **G38** | *Dactylorhiza majalis* 98.72%  *Platanthera mandarinorum* 98.72%  **NO^[2]^** |

NO^[1]^: more than one reference sequence at top Bit-Score (at least 99.5 %)

NO^[2]^: all reference sequences at top Bit-score lower than 99.5%
