# Supplementary material for: DNA barcoding of native Caucasus herbal plants: potentials and limitations in complex groups and implications for phylogeographic patterns
Source: Biodivers Data J. 2021 Jan 27;9:e61333. doi: 10.3897/BDJ.9.e61333 (PMC7858560; doi:10.3897/BDJ.9.e61333)
Supplement: Supplementary material 4 — Results of BLAST species identification test for asterid ITS [file bdj-09-e61333-s004.docx]

SuppTab4: Results of BLAST species identification test for asterid ITS

| Sample | Top Bit-score |
| --- | --- |
| **P1** | *Centaurea nogmovii* 99.84 %  *Centaurea cabardensis* 99.18 %  **YES** |
| **P2** | *Centaurea nogmovii* 99.35 %  *Centaurea cabardensis* 98.69 %  **NO^[2]^** |
| **P3** | *Psephellus hadimensis* 100 %  *Psephellus xanthocephalus* 99.05 %  **YES** |
| **P4** | *Centaurea nogmovii* 99.51 %  *Centaurea cabardensis* 98.86 %  **YES** |
| **P5** | *Centaurea nogmovii* 99.84 %  *Centaurea cabardensis* 99.18 %  **YES** |
| **P6** | *Psephellus hadimensis* 100 %  *Psephellus xanthocephalus* 99.05 %  **YES** |
| **P7** | *Centaurea nogmovii* 100 %  *Centaurea cabardensis* 99.02 %  **YES** |
| **P8** | *Centaurea nogmovii* 100 %  *Centaurea cabardensis* 99.02 %  **YES** |
| **P9** | *Centaurea nogmovii* 99.84 %  *Psephellus pulcherrimus* 98.58 %  **YES** |
| **A1** | *Leucanthemum vulgare* 99.84%  *Leucanthemum gaudinii* 99.84 %  **NO^[1]^** |
| **A2** | *Leucanthemum ageratifolium* 99.36%  *Leucanthemum vulgare* 99.36 %  **NO^[2]^** |
| **A3** | *Leucanthemum vulgare* 99.84%  *Leucanthemum gaudinii* 99.84 %  **NO^[1]^** |
| **A4** | *Leucanthemum vulgare* 99.03%  *Leucanthemum gaudinii* 99.03 %  **NO^[2]^** |
| **A5** | *Leucanthemum vulgare* 99.52%  *Leucanthemum gaudinii* 99.52 %  **NO^[1]^** |
| **A6** | *Leucanthemum vulgare* 99.36%  *Leucanthemum gaudinii* 99.36 %  **NO^[2^**^]^ |
| **A7** | *Bellis pusilla* 99.68 %  *Bellis bernardii* 99.20%  **YES** |
| **A8** | *Bellis pusilla* 99.68 %  *Bellis bernardii* 99.20 %  **YES** |
| **A9** | *Hypochaeris radicata* 100 %  *Taraxacum alpinum* 99.84 %  **YES** |
| **A10** | *Leontodon hispidus* 99.84 %  *Leontodon taraxacoides* 99.36 %  **YES** |
| **A11** | *Taraxacum obtusifrons* 99.69 %  *Taraxacum mongolicum* 99.69 %  **NO^[1]^** |
| **A12** | *Tanacetum coccineum* 100 %  *Tanacetum abrotanifolium* 98.87 %  **YES** |
| **A13** | *Senecio vernalis* 99.84 %  *Senecio rupestris* 99.69 %  **YES** |
| **A14** | *Symphyotrichum novae-angliae* 99.84 %  *Symphyotrichum adnatum* 98.71%  **YES** |
| **A15** | *Bellis pusilla* 99.04 %  *Bellis bernardii* 98.55%  **NO^[2]^** |

NO^[1]^: more than one reference sequence at top Bit-Score (at least 99.5 %)

NO^[2]^: all reference sequences at top Bit-score lower than 99.5%
