# Supplementary material for: DNA barcoding of native Caucasus herbal plants: potentials and limitations in complex groups and implications for phylogeographic patterns
Source: Biodivers Data J. 2021 Jan 27;9:e61333. doi: 10.3897/BDJ.9.e61333 (PMC7858560; doi:10.3897/BDJ.9.e61333)
Supplement: Supplementary material 6 — Results of BLAST species identification test for asterid accD [file bdj-09-e61333-s006.docx]

SuppTab6: Results of BLAST species identification test for asterid *accD*

| Sample | Top Bit-score |
| --- | --- |
| **P1** | *Eclipta alba* 99.49 %  *Sphagneticola calendulacea* 99.49 %  **NO^[1]^** |
| **P2** | *Eclipta alba* 99.49 %  *Sphagneticola calendulacea* 99.49 %  **NO^[1]^** |
| **P3** | *Eclipta alba* 99.49 %  *Sphagneticola calendulacea* 99.49 %  **NO^[1]^** |
| **P4** | *Eclipta alba* 99.49 %  *Sphagneticola calendulacea* 99.49 %  **NO^[1]^** |
| **P5** | *Eclipta alba* 99.49 %  *Sphagneticola calendulacea* 99.49 %  **NO^[1]^** |
| **P6** | *Eclipta alba* 99.49 %  *Sphagneticola calendulacea* 99.49 %  **NO^[1]^** |
| **P7** | *Eclipta alba* 99.49 %  *Sphagneticola calendulacea* 99.49 %  **NO^[1]^** |
| **P8** | *Eclipta alba* 99.49 %  *Sphagneticola calendulacea* 99.49 %  **NO^[1]^** |
| **P9** | *Eclipta alba* 99.49 %  *Sphagneticola calendulacea* 99.49 %  **NO^[1]^** |
| **A1** | *Artemisia stolonifera* 100 %  *Artemisia sieversiana* 100 %  *Leucanthemum virgatum* 100 %  *Tanacetum coccineum* 100 %  **NO^[1]^** |
| **A2** | *Artemisia stolonifera* 100 %  *Artemisia sieversiana* 100 %  *Leucanthemum virgatum* 100 %  *Tanacetum coccineum* 100 %  **NO^[1]^** |
| **A3** | *Artemisia stolonifera* 100 %  *Artemisia sieversiana* 100 %  *Leucanthemum virgatum* 100 %  *Tanacetum coccineum* 100 %  **NO^[1]^** |
| **A4** | *Artemisia stolonifera* 99.49 %  *Artemisia sieversiana* 99.49 %  *Leucanthemum virgatum* 99.49 %  *Tanacetum coccineum* 99.49 %  **NO^[1]^** |
| **A5** | *Artemisia stolonifera* 100 %  *Artemisia sieversiana* 100 %  *Leucanthemum virgatum* 100 %  *Tanacetum coccineum* 100 %  **NO^[1]^** |
| **A6** | *Artemisia stolonifera* 100 %  *Artemisia sieversiana* 100 %  *Leucanthemum virgatum* 100 %  *Tanacetum coccineum* 100 %  **NO^[1]^** |
| **A7** | *Heteroplexis incana* 98.97 %  *Aster hypoleucus* 98.97 %  **NO^[2]^** |
| **A8** | *Heteroplexis incana* 98.97 %  *Aster hypoleucus* 98.97 %  **NO^[2]^** |
| **A9** | *Taraxacum officinale* 100 %  *Taraxacum kok-saghyz* 100 %  **NO^[1]^** |
| **A10** | *Sonchus asper* 100 %  *Sonchus oleraceus* 100 %  **NO^[1]^** |
| **A11** | *Sonchus asper* 98.94 %  *Sonchus oleraceus* 98.94 %  *Taraxacum officinale* 98.41%  **NO ^[2]^** |
| **A12** | *Artemisia stolonifera* 100 %  *Artemisia sieversiana* 100 %  *Tanacetum coccineum* 100 %  **NO^[1]^** |
| **A13** | *Senecio schweinfurthii* 100 %  *Senecio moorei* 100 %  *Senecio vulgaris* 99.49 %  **NO^[1]^** |
| **A14** | *Heteroplexis incana* 100%  *Aster hypoleucus* 100 %  **NO^[1]^** |
| **A15** | *Artemisia stolonifera* 100 %  *Artemisia sieversiana* 100 %  *Leucanthemum virgatum* 100 %  *Tanacetum coccineum* 100 %  **NO^[1]^** |

NO^[1]^: more than one reference sequence at top Bit-Score (at least 99.5 %)

NO^[2]^: all reference sequences at top Bit-score lower than 99.5%
