# Supplementary material for: DNA barcoding of native Caucasus herbal plants: potentials and limitations in complex groups and implications for phylogeographic patterns
Source: Biodivers Data J. 2021 Jan 27;9:e61333. doi: 10.3897/BDJ.9.e61333 (PMC7858560; doi:10.3897/BDJ.9.e61333)
Supplement: Supplementary material 7 — Highest BLAST match of ITS sequences for the samples of Orchidaceae examined in this study [file bdj-09-e61333-s007.doc]

Supplementary table 7. Highest BLAST match of ITS sequences for the samples of Orchidaceae examined in this study.

Sample	Highest BLAST match (Acces. num.)	Origin	Reference	
G14	Anacamptis pyramidalis [MT179754.1]	Denmark٭	Hartvig, 2020 Unpublished	
	Anacamptis pyramidalis [MT179741.1]	Denmark٭	Hartvig, 2020 Unpublished	
	Anacamptis pyramidalis [KY512500.1]	Turkey	Sungu Seker et al., 2017	
	Anacamptis pyramidalis [KU931734.1]	Iran	Ghorbani et al., 2016	
	Anacamptis pyramidalis [KU931733.1]	Iran	Ghorbani et al., 2016	
	Anacamptis pyramidalis [KU931732.1 ]	Iran	Ghorbani et al., 2016	
	Anacamptis pyramidalis [KU931730.1 ]	Iran	Ghorbani et al., 2016	
	Anacamptis pyramidalis [AY364870.1]	Spain	Bernardos  et al., 2003 Unpublished	
G7, G9,G12	Anacamptis pyramidalis [Z94061.1]	Italy	Aceto et al., 1999	
G15	Cephalanthera damasonium [AF521060.1]	UK٭	van den Berg et al. 2005	
	Cephalanthera damasonium [AY146446.1]	Austria٭	Moscone et al., 2007  	
	Cephalanthera damasonium [AY833027.1]	France٭	Julou et al., 2005	
	Cephalanthera damasonium [MT179737.1]	Denmark٭	Hartvig, 2020 Unpublished	
G19	Dactylorhiza maculata [MH016591.1]	Russia	Kirillova et al. 2018	
	Dactylorhiza maculata [MT179743.1]	Denmark٭	Hartvig, 2020 Unpublished	
	Dactylorhiza maculata [AY704973.1]	Switzerland٭	van der Niet et al., 2005	
	Dactylorhiza maculata [MT179756.1]	Denmark٭	Hartvig, 2020 Unpublished	
	Dactylorhiza maculata [MH016580.1]	Russia	Kirillova et al. 2018	
	Dactylorhiza maculata [AY699479.1]	Belgium٭	Devos et al., 2005	
	Dactylorhiza maculata [AY699462.1]	Belgium٭	Devos et al., 2005	
	Dactylorhiza maculata [AY699458.1]	Belgium٭	Devos et al., 2005	
	Dactylorhiza maculata [AY699450.1]	Belgium٭	Devos et al., 2005	
	Dactylorhiza maculata [MH016579.1]	Russia	Kirillova et al. 2018	
	Dactylorhiza maculata [DQ022868.1]	Sweden	Pillon et al., 2006	
	Gymnadenia conopsea [Z94068.1]	Italy٭	Aceto et al., 1999	
	Gymnadenia conopsea [JF414025.1]	Germany٭	Stark et al., 2011	
	Gymnadenia conopsea [FJ751758.1]	Czech Republic	Jersakova, Schodelbauerova, 2010	
	Gymnadenia conopsea [FJ751757.1]	Czech Republic	Jersakova, Schodelbauerova, 2010	
	Gymnadenia conopsea [JQ768210.1]	Russia	Efimov, 2013	
G18	Gymnadenia conopsea [JQ768209.1]	Russia	Efimov, 2013	
	Gymnadenia conopsea [JQ768207.1]	Russia	Efimov, 2013	
	Gymnadenia conopsea [JQ768205.1]	Russia	Efimov, 2013	
	Gymnadenia conopsea [JQ768204.1]	Russia	Efimov, 2013	
	Gymnadenia conopsea [JQ730034.1]	Russia	Efimov, 2013	
	Gymnadenia conopsea [JN696449.1]	China٭	Jin et al., 2011
Unpublished	
	Gymnadenia conopsea [DQ351281.1]	UK٭	Bateman
 Et al., 2006	
	Gymnadenia conopsea [AY704974.1]	Switzerland٭	van der Niet et al., 2005	
	Gymnadenia conopsea [KY512498.1]	Turkey	Sungu Seker et al., 2017	
	Gymnadenia conopsea [DQ022888.1]	France	Pillon et al., 2006	
G5, G37	Orchis adenocheila [KU931696.1 ]	Iran	Ghorbani et al., 2016	
G38	Orchis adenocheila [KU931697.1]	Iran	Ghorbani et al., 2016	
	Orchis adenocheila [KU931695.1]	Iran	Ghorbani et al., 2016	
	Orchis adenocheila [KU931694.1]	Iran	Ghorbani et al., 2016	
	Orchis mascula [Z94088.1]	Italy٭	Aceto et al., 1999	
	Orchis mascula [KU931688.1]	Iran	Ghorbani et al., 2016	
	Orchis mascula [KU931687.1]	Iran	Ghorbani et al., 2016	
G11	Orchis mascula [KU931686.1]	Iran	Ghorbani et al., 2016	
G10	Orchis mascula [KU931685.1]	Iran	Ghorbani et al., 2016	
	Orchis mascula [KU931684.1]	Iran	Ghorbani et al., 2016	
	Orchis mascula [KU931683.1]	Iran	Ghorbani et al., 2016	
	Orchis mascula [AY351379.1]	Spain	Bernardos et al., 2004	
	Orchis mascula subsp. pinetorum [KU697371.1] 	Turkey٭	Dizkirici et al., 2017	
G36, G35, G31, G29, G27, G4, G3, G2	Orchis militaris [AY014548.1]	Switzerland٭	Soliva et al., 2001
	
	Orchis militaris [AY699977.1]	Spain	Bernardos et al., 2004	
	Orchis militaris [Z94090.1]	Italy٭	Aceto et al., 1999	
	Orchis purpurea  [AY364882.1]	Spain	Bernardos et al., 2004	
	Orchis purpurea  [MT179755.1]	Denmark٭	Hartvig, 2020	
	Orchis purpurea  [MT179742.1] 	Denmark٭	Hartvig, 2020	
	Orchis purpurea  [Z94103.1]	Italy٭	Aceto et al., 1999	
G6	Orchis simia [Z94108.1]	Italy٭	Aceto et al., 1999	
	Orchis simia [KY584050.1]	Turkey٭	Sungu Seker et al., 2017	
	Orchis simia [KU697369.1]	Turkey٭	Dizkirici et al., 2017	
	Orchis simia [KU931692.1]	Iran	Ghorbani et al., 2016	
	Orchis simia [KU931691.1]	Iran	Ghorbani et al., 2016	
	Orchis simia [KU931690.1]	Iran	Ghorbani et al., 2016	
	Orchis simia [KU931689.1]	Iran	Ghorbani et al., 2016	
G17	Ophrys sphegodes [KU931720.1]	Iran	Ghorbani et al., 2016	
	Ophrys sphegodes [KU931706.1]	Iran	Ghorbani et al., 2016	
	Ophrys sphegodes [KU931703.1]	Iran	Ghorbani et al., 2016	
	Ophrys sphegodes [AY699974.1]	Spain	Bernardos et al., 2004	
	Ophrys sphegodes [AY014542.1]	Switzerland٭	Soliva et al., 2001	
	Ophrys sphegodes [AJ973255.1]	Hungary	Gulyas et al., 2005	
	Ophrys sphegodes [AM711739.1]	UK	Devey et al., 2008	
G1, G16	Platanthera chlorantha [Z94118.1]	Italy٭	Aceto et al., 1999	
	Platanthera chlorantha [MF944373.1]	China٭	Jin et al., 2017	
	Platanthera chlorantha [KU931701.1]	Iran	Ghorbani et a;l., 2016	
	Platanthera chlorantha [KU931699.1]	Iran	Ghorbani et a;l., 2016	
	Platanthera chlorantha [KY007623.1]	Germania	Durka et al., 2017	
	Platanthera chlorantha [KJ460085.1]	China٭	Jin et al., 2014	
	Platanthera chlorantha [MT179753.1]	Denmark٭	Hatvig, 2020
Unpublished	
	Platanthera chlorantha [MT179736.1]	Denmark٭	Hatvig, 2020
Unpublished	
	Platanthera chlorantha [KU931702.1]	Iran	Ghorbani et al., 2016	
	Platanthera chlorantha [KY007624.1]	Germania	Durka et al., 2017	
	Platanthera chlorantha [KT338767.1]	Republic
of Korea	Choi et al., 2015	
	Platanthera chlorantha [AY704975.1]	Switzerland٭	van der Niet et al., 2005	
	Orchis adenocheila [KU931693]	
Platanthera chlorantha [KU931700] 
Platanthera chlorantha [KY512507]
Anacamptis pyramidalis [KU931733]
Cephalanthera damasonium [AY833027]	Azerbaijan 
Azerbaijan
Turkey 
Iran 
France 	Ghorbani et al., 2016
Ghorbani et al., 2016
Sungu Seker et al., 2018
Ghorbani et al., 2016
Julou et al., 2005


	
Note: (٭) points to the sequences with unclear origin. For these sequences the origin (country) of organisms is not specified and in that case the origin is considered that of the authors. 

Note: Reference

Aceto S., Caputo P., Cozzolino S., Gaudio L., Moretti A. 1999; Phylogeny and evolution in Orchis and allied genera based on ITS DNA variation: morphological gaps and molecular continuity; Molecular Phylogenetics and Evolution Vol. 13, No. 1, October, pp. 67–76
Bateman R.M., Rudall P.J., James K.E. 2006. Phylogenetic context, generic affinities and evolutionary origin of the enigmatic Balkan orchid Gymnadenia frivaldii Hampe ex Griseb. Taxon 55 (1), 107-118
Bernardos S., Tyteca D., Revuelta J.L., Amich F. 2004. A new endemic species of Epipactis (Orchidaceae) from north-east Portugal;  Bot. J. Linn. Soc. 145 (2), 239-249
Choi S.A., So J.H., Eum S.M., Lee N.S. Evaluation of Internal Transcribed Spacer Region as DNA Barcode and phylogenetic tool for Orchidaceae from Korea (Unpublished)
Devey D.S., Bateman R.M., Fay M.F., Hawkins J.A. 2008. Friends or relatives? Phylogenetics and species delimitation in the controversial European orchid genus Ophrys; Ann. Bot. 101 (3), 385-402
Devos N., Oh S.H., Raspe O.,Jacquemart A.L., Manos P.S. 2005; Nuclear ribosomal DNA sequence variation and evolution of spotted
marsh-orchids (Dactylorhizamaculata group); Mol. Phylogenet. Evol. 36 (3), 568-580
Dizkirici A., Isler S., Yigit O. 2017. Molecular phylogenetics of some Orchis species (Orchidaceae) native to Turkey using nuclear and chloroplast DNA sequences; J Sci 30(2): 35-43
Durka W., Baum A., Michalski S.G., Baum H. 2017. Darwin's legacy in Platanthera: are there more than two species in
The Platanthera bifolia/ chlorantha group? Plant Systematics and Evolution volume 303: 419–431
Efimov P.G. 2013. Sibling species of fragrant orchids (Gymnadenia: Orchidaceae, Magnoliophyta) in Russia. Russian Journal of Genetics,  49: 299–309(2013)
Ghorbani A., Gravendeel B., Selliah S., Zarre,S., de Boer H. 2016. DNA barcoding of tuberous Orchidoideae: a resource for identification of orchids used in Salep. Mol Ecol Resour17, 342–352
Gulyas G., Sramko G., Molnar V.A., Rudnoy S., Illyes Z., Balazs T., Bratek Z. 2005. Nuclear ribosomal DNA ITS paralogs as evidence of recent interspecific hybridization in the genus Ophrys (Orchidaceae);ActaBiolCracSer Bot 47 (2), 61-67
Jersakova J., Schodelbauerova I. 2010. Absence of pollinator-mediated premating barriers in ploidy-mixed
populations of Gymnadeniaconopseas.l. (Orchidaceae) ; Evolutionary Ecology volume 24, 1199–1218
Jin X., Li D., Xiang X., Lai Y., Shi X. 2011. Nujiangia (Orchidaceae: Orchideae): A new genus from Yunnan, China. 
DOI: 10.1111/j.1759-6831.2011.00167.x
Julou T., Burghardt B., Gebauer G., Berveiller D., Damesin C., Selosse M.A. 2005. Mixotrophy in orchids: insights from a comparative study of green individuals and nonphotosynthetic individuals of Cephalantheradamasonium; New Phytol. 166 (2), 639-653
Hartvig I. 2020 (Unpublished) Barcoding of Danish orchids; 
Kirillova I.A., Kirillov D.V., Shadrin D.M. (2018); Molecular and morphological approaches to studying the Dactylorhiza
genus in the Komi Republic; Vestn Tomsk GosUniv Biol 43, 44-65
Moscone E.A., Pedrosa A., Samuel R., Pointner R., Schwarzacher T., Schweizer D. 2002. A molecular and cytogenetic study of phylogenetic relationships in Cephalanthera (Orchidaceae). (Unpublished)
Pillon,Y., Fay,M.F., Shipunov,A.B. and Chase,M.W.; 2006; Species diversity versus phylogenetic diversity: A practical study
in the taxonomically difficult genus Dactylorhiza (Orchidaceae); Biol. Conserv. 129 (1), 4-13
Soliva M., Kocyan A., Widmer A. 2001. Molecular phylogenetics of the sexually deceptive orchid genus Ophrys (Orchidaceae) based on nuclear and chloroplast DNA sequences. Mol. Phylogenet. Evol. 20 (1), 78-88
Stark C., Michalski S.G., Babik W., Winterfeld G., Durka W.; 2011; Strong genetic differentiation between Gymnadeniaconopsea and G. densiflora despite morphological similarity; Plant Syst. Evol. 293 (1-4), 213-226
Sungu Seker,S., Akbulut,M.K., Senel,G. and ErgenAkcin,O. 2017. An Integrative Approach to Anatomy, Morphology, Micromorphology and
Molecular Phylogeny of Epidendroid and Orchidoid Species (Orchidaceae); Nordic journal of Botany, 2018  36 (7)
van den Berg C., Goldman D.H., Freudenstein, .V., Pridgeon A.M., Cameron,K.M., Chase,M.W. 2005; An overview of the phylogenetic relationships within Epidendroideae inferred from multiple DNA regions and recircumscription of Epidendreae and Arethuseae (Orchidaceae). Am. J. Bot. 92 (4), 613-624.
van der Niet T., Linder H.P., Bytebier B., Bellstedt D.U. 2005. Molecular Markers Reject Monophyly of the Subgenera of Satyrium
(Orchidaceae);Syst. Bot. 30 (2), 263-274
