# Supplementary material for: DNA barcoding of native Caucasus herbal plants: potentials and limitations in complex groups and implications for phylogeographic patterns
Source: Biodivers Data J. 2021 Jan 27;9:e61333. doi: 10.3897/BDJ.9.e61333 (PMC7858560; doi:10.3897/BDJ.9.e61333)
Supplement: Supplementary material 8 — Highest BLAST match of ITS sequences for the samples of Asteraceae examined in this study [file bdj-09-e61333-s008.doc]

Supplementary table 8. Highest BLAST match of ITS sequences for the samples of Asteraceae examined in this study

Sample 			Highest  BLAST match (Acces. numb.)	Origin	Reference	
P1-P8			Centaurea nogmovii  [JX274510]	Russia	Shardin et al., 2012. Unpiblished	
			Centaurea trinervia [AJ304790]	Germany٭	Hellwig F.H. 2004	
			Centaurea cabardensis [JX274509.1]	Russia	Shardin et al.  2012. Unpiblished	
P3-P6			Psephellus xanthocephalus [AY829445.1]	Spain٭	Susanna et al. 2006.	
			Psephellus hadimensis [KX158189.1]	Turkey٭	Uysal, 2016	
			Leontodon hispidus [KX643619.1]	Slovakia٭	Slovak et al., 2016 Unpiblished	
A10			Leontodon hispidus [KT249914.1]	Romania	Choi et al., 2015	
			Leontodon hispidus [JF801910.1]	Germany٭	Enke et al., 2012	
			Leontodon hispidus [DQ451771.1]	Austria٭	Samuel et al., 2006	
			Leontodon hispidus [DQ451770.1]	Austria٭	Samuel et al., 2006	
			Leontodon hispidus [AF528485.1]	Austria٭	Samuel et al., 2003	
A14			Symphyotrichum novae-angliae [GU818492.1]	USA	Pelser et al., 2010	
A14			Symphyotrichum novae-angliae [JQ360398.1]	USA٭	Morgan, Holland, 2012	
			Leucanthemum vulgare [MK481551.1]	Germany	Wagner et al., 2019	
			Leucanthemum vulgare [EF577315.1]	China٭	Zhao et al., 2010	
			Leucanthemum vulgare [MK481420.1]	France	Wagner et al., 2019	
			Leucanthemum gallaecicum  [MK481560.1]	Spain	Wagner et al., 2019	
			Leucanthemum pyrenaicum [MK481548.1]	Spain	Wagner et al., 2019	
			Leucanthemum pyrenaicum [MK481547.1]	Slovakia	Wagner et al., 2019	
			Leucanthemum cacuminis [MK481415.1]	Spain	Wagner et al., 2019	
			Leucanthemum ligusticum [MK481412.1]	Italy	Wagner et al., 2019	
			Leucanthemum pluriflorum [MK481410.1]	Spain	Wagner et al., 2019	
			Bellis pusilla [KP175223.1]	Italy	Park, 2014 
Unpublished	
A7-8, 15			Bellis margaritifolia [AF492846.1]	Italy,  Sicily	Fiz et al., 2002	
			Bellis perennis [KX446803.1 ]	China٭	Luo et al., 2016	
A12			Tanacetum coccineum [KY397500.1]	Australia٭	Jayasena et al., 2017	
			Tanacetum coccineum [AB608333.1]	Iran٭	Sonboli et al., 2011
Unpublished	
A13			Senecio vernalis [KT249848.1]	Germany	Choi, Thines, 2015	
			Senecio vernalis [JN789911.1]	Israel	Palser et al., 2012	
			Senecio vernalis [KT249850.1]	Germany	Choi, Thines, 2015	
			Senecio vernalis [AJ400806.1]	Germany	Comes, Abbot,2001	
			Senecio vernalis [KT249846.1]	Germany	Choi, Thines, 2015	
A9			Taraxacum alpinum [AJ633289.1]	Romania	Gemeinholzer, Bachmann, 2004
Unpublished	
A11			Taraxacum officinale [KT249884.1]	Germany	Choi, Thines, 2015	
			Taraxacum officinale [KT249883.1]	Germany	Choi, Thines, 2015	
			Taraxacum officinale [JQ230979.1]	India٭	Malik, Babbar, 2001
Unpublished	
			Taraxacum officinale [AY548211.1]	South Korea٭	Choi et al., 2004	
			Taraxacum officinale [MG519306.1]	China٭	Akram, Jafri, …. 
Unpublished	
			Taraxacum officinale [KY860926.1]	USA٭	Anderson, 2017	
			Taraxacum officinale [AB766235.1]	France	Shibaike et al., 2002	
			Taraxacum officinale [AJ633290.1]	Romania	Gemeinholzer, Bachmann, 2004
Unpublished	

Note: (٭) points to the sequences with unclear origin. For these sequences the origin (country) of organisms is not specified and in that case the origin is considered that of the authors. 

Note: Reference
Akram,W.,Jafri, M.J. (Unpublished) Evolution of Taraxacum;
Anderson, J.R. 2017  (Unpublished)
Choi,Y.J., Thines,M. 2015; Host Jumps and Radiation, Not Co‐Divergence Drives Diversification of Obligate Pathogens. A Case Study in Downy Mildews and Asteraceae; PLoS ONE 10 (7)
Comes H.P., Abbott R.J. 2001; Molecular phylogeography, reticulation, and lineage sorting in Mediterranean Senecio sect. Senecio (Asteraceae); Evolution 55 (10), 1943-1962
Enke N., Gemeinholzer B., Zidorn Ch. 2012. Molecular and phytochemical systematics of the subtribeHypochaeridinae (Asteraceae, Cichorieae. Org Divers Evol 12:1–16.
Fiz et al., 2002: Fiz,O., Valcarcel,V.,Vargas,P; 2002; Phylogenetic position of Mediterranean Astereae and character
evolution of daisies (Bellis, Asteraceae) inferred from nrDNA ITS sequences; Mol. Phylogenet. Evol. 25 (1): 157-171
Gemeinholzer B., Bachmann,K. (Unpublished); Molecular systematics of the Lactuceae (Asteraceae: Cichoriaceae): combined nuclear and chloroplast data. 
Hellwig,F.H. 2004 Centaureinae (Asteraceae) in the Mediterranean – history of ecogeographical radiation. Plant Systematics and Evolution volume 246:37–162.
Jayasena et al., 2017: Jayasena,A.S., Fisher,M.F., Panero,J.L., Secco,D., Bernath-Levin,K., Berkowitz,O., Taylor,N.L., Schilling,E.E.,  Whelan,J., Mylne,J.S.;2017; Stepwise evolution of a buried inhibitor peptide over 45 million years; Molecular Biology and Evolution, 34 (6): 1505–1516
Luo et al., 2016: Luo,C., Chen,D., Cheng,X., Zhao,H. and Huang,C.; 2017; Genome size estimations in Chrysanthemum and correlations with  molecular phylogenies; Genetic Resources and Crop Evolution, 64: 1451–1463
Malik, Babbar, 2001 (Unpublished) DNA barcoding of Indian medicinal plants
Morgan, Holland, 2012 Systematics of Symphyotrichinae (Asteraceae: Astereae): Disagreements between Two Nuclear Regions Suggest a Complex Evolutionary History. Syst. Bot. 37 (3), 818-832 (2012)
Park, 2014 (Unpublished) Close Relatives Make Bad Neighbors in a Reciprocal Test of Darwinsm Naturalization Hypothesis in the Mediterranean 
Pelser et al., 2010: Pelser P.B., Abbott R.J., Comes H.P., Milton J.J., Moller M., Looseley M.E., Cron G.V., Barcelona J.F., Kennedy A.H., Watson L.E., Barone, R., Hernandez,F., Kadereit J.W. 2012; The genetic ghost of an invasion past: colonization and extinction revealed by historical hybridization in Senecio; Mol. Ecol. 21 (2), 369-387
Pieter B., Pelser  Aaron H. Kennedy  Eric J. Tepe  Jacob B. ShidlerBertilNordenstam  Joachim W. Kadereit  Linda E. Watson; 2010; Patterns and causes of incongruence between plastid and nuclear Senecioneae (Asteraceae) phylogenies; Am. J. Bot. 97 (5)
Samuel R., Gutermann W., Stuessy T.F., Ruas C.F., Lack H.-W., Tremetsberger K., Talavera S., Hermanowski B., Ehrendorfer F. 2006; Molecular Phylogenetics Reveals Leontodon (Asteraceae, Lactuceae) to Be Diphyletic; American Journal of Botany 93(8) pp 1193–1205.
Shardin et al.  2012. (Unpiblished) Shadrin,D.M., Pylina,Y.I., Druz,Y.I., Volodina,S.O., Chadin,I.F.  and Volodin,V.V.; Molecular Phylogeny and Chemotaxonomy of ecdysteroid-containing plants of families CaryophyllaceaeJuss. and Asteraceae Dumort
Shibaike,H., Morita and T.; 2002; Hybridization between European and Asian dandelions (Taraxacum section Ruderalia and section Mongolica) 3. Natural hybrids in Japan detected by nuclear DNA marker; J Plant Res 115:321–328
Slovak et al., 2016 (Unpiblished): Slovak,M., Kucera,J., Lack,H.W., Zifer-Berger,J., Plecenikova,A., Zaveska,E. and Vdacny,P.; Reaching the southern hemisphere: paleoclimatic oscillations and shifts in intrinsic traits triggered bipolar disjunction in the genus Picris (Compositae)
Sonboli,A., KazempourOsaloo,S., Valles,J. and Oberprieler,C.; 2011; Systematic status and phylogenetic relationships of the enigmatic TanacetumparadoxumBornm. (Asteraceae, Anthemideae): evidences from nrDNA ITS, micromorphological, and cytological data; Plant Systematics and Evolution volume 292: 85–93
Susanna A., Garcia-Jacas,N., Hidalgo,O.,Vilatersana,R. and Garnatje,T.; 2006.The Cardueae (Compositae) revisited: insights from a combined ITS,
trnL-trnF and matK nuclear and chloroplast DNA analysis.ANN. MISSOURI BOT. GARD. 93: 150–171.
Uysal T. 2008; A new species of Centaurea (Asteraceae) from Turkey; AnnalesbotaniciFennicivol 45.
Young-Joon Choi, Marco Thines; 2015; Host Jumps and Radiation, Not Co‐Divergence Drives Diversification of Obligate Pathogens. A Case Study in Downy Mildews and Asteraceae;PLoS ONE 10 (7)
Wagner F.,  Ott T.,  Zimmer C., Reichhart V., Vogt R., Oberprieler Ch.;  2019; At the crossroads towards polyploidy': genomic divergence and extent of homoploid hybridization are drivers for the formation of the ox-eye daisy polyploid complex (Leucanthemum , Compositae-Anthemideae); New Phytol.
Zhao et al., 2010:  Zhao H.-B., Chen F.-D., Chen S.-M., Wu G.-S., Guo W.-M.; 2010; Molecular phylogeny of Chrysanthemum, Ajania and its allies (Anthemideae, Asteraceae) as inferred from nuclear ribosomal ITS and chloroplast trnL-F IGS sequences; Plant Syst. Evol. 284 (3-4): 153-169
